# Supplementary material for: Neuromusculoskeletal modeling of spasticity: A scoping review
Source: PLoS One. 2025 May 14;20(5):e0320153. doi: 10.1371/journal.pone.0320153 (PMC12077711; doi:10.1371/journal.pone.0320153)
Supplement: S2 Table — (DOCX) [file pone.0320153.s002.docx]

**S2 Table. Search strategy used for each of the databases.**

| **Database** | **Select fields** | **Search Strategy** |
| --- | --- | --- |
| **Pubmed** | Title/Abstract and Other Term | ("muscle spasticity"[Title/Abstract] AND "neuro-musculoskeletal models"[Title/Abstract] OR "neuro-musculoskeletal model"[Title/Abstract] OR "neuromusculoskeletal models"[Title/Abstract] OR "neuromusculoskeletal model"[Title/Abstract] OR "neuro-musculoskeletal modeling"[Title/Abstract] OR "neuromusculoskeletal modeling"[Title/Abstract] OR "neuro-musculoskeletal modelling"[Title/Abstract] OR "neuromusculoskeletal modelling"[Title/Abstract] OR "neuromusculoskeletal simulation"[Title/Abstract] OR "neuro-musculoskeletal simulation"[Title/Abstract]) OR ("muscle spasticity"[Other Term] AND "neuro-musculoskeletal models"[Other Term] OR "neuro-musculoskeletal model"[Other Term] OR "neuromusculoskeletal models"[Other Term] OR "neuromusculoskeletal model"[Other Term] OR "neuro-musculoskeletal modeling"[Other Term] OR "neuromusculoskeletal modeling"[Other Term] OR "neuro-musculoskeletal modelling"[Other Term] OR "neuromusculoskeletal modelling"[Other Term] OR "neuromusculoskeletal simulation"[Other Term] OR "neuro-musculoskeletal simulation"[Other Term]) |
| **Engineering Village (Compendex)** | Expert search | "muscle spasticity" wn ab AND "neuro-musculoskeletal models" OR "neuro-musculoskeletal model" OR "neuromusculoskeletal models" OR "neuromusculoskeletal model" OR "neuro-musculoskeletal modeling" OR "neuromusculoskeletal modeling" OR "neuro-musculoskeletal modelling" OR "neuromusculoskeletal modelling" OR "neuromusculoskeletal simulation" OR "neuro-musculoskeletal simulation" wn ab OR "muscle spasticity" wn ti AND "neuro-musculoskeletal models" OR "neuro-musculoskeletal model" OR "neuromusculoskeletal models" OR "neuromusculoskeletal model" OR "neuro-musculoskeletal modeling" OR "neuromusculoskeletal modeling" OR "neuro-musculoskeletal modelling" OR "neuromusculoskeletal modelling" OR "neuromusculoskeletal simulation" OR "neuro-musculoskeletal simulation" wn ti OR "muscle spasticity" wn fl AND "neuro-musculoskeletal models" OR "neuro-musculoskeletal model" OR "neuromusculoskeletal models" OR "neuromusculoskeletal model" OR "neuro-musculoskeletal modeling" OR "neuromusculoskeletal modeling" OR "neuro-musculoskeletal modelling" OR "neuromusculoskeletal modelling" OR "neuromusculoskeletal simulation" OR "neuro-musculoskeletal simulation" wn fl |
| **IEEE Xplore** | Abstract, Document Title and Author Keywords | "muscle spasticity" AND "neuro-musculoskeletal models" OR "neuro-musculoskeletal model" OR "neuromusculoskeletal models" OR "neuromusculoskeletal model" OR "neuro-musculoskeletal modeling" OR "neuromusculoskeletal modeling" OR "neuro-musculoskeletal modelling" OR "neuromusculoskeletal modelling" OR "neuromusculoskeletal simulation" OR "neuro-musculoskeletal simulation" |
| **Science Direct** | Title, abstract or author-specified keywords | "muscle spasticity" AND "neuro-musculoskeletal model" OR "neuromusculoskeletal model" OR "neuro-musculoskeletal modeling" OR "neuromusculoskeletal modeling" OR "neuro-musculoskeletal modelling" OR "neuromusculoskeletal modelling" OR "neuromusculoskeletal simulation" OR "neuro-musculoskeletal simulation" |
