# Supplementary material for: Neuromusculoskeletal modeling of spasticity: A scoping review
Source: PLoS One. 2025 May 14;20(5):e0320153. doi: 10.1371/journal.pone.0320153 (PMC12077711; doi:10.1371/journal.pone.0320153)
Supplement: S3 Table — (DOCX) [file pone.0320153.s003.docx]

**S3 Table. Physiological plausibility score for the developed models according to the investigated tasks, target muscles and/or joints, and evaluated groups.**

| **Authors** | **Comparision with experimental data** | **Task** | **Target muscles or joints** | **Feedback Model** | **Group** | **NF** | **i** | **ii** | **iii** | **iv** | **v** | **Sum of model in each condition** | **Sum of the points of the model** | **Physiological plausibility Score** |
| --- | --- | --- | --- | --- | --- | --- | --- | --- | --- | --- | --- | --- | --- | --- |
| van der Krogt et al., 2016 | RMSE | Passive stretching with controlled duration executed by a practitioner | Hamstrings at slow velocity | MSV | SG | 4 | 0 | 0 | 0 | 0 | 1 | 1 | 4 | **1** |
| van der Krogt et al., 2016 |  | Passive stretching with controlled duration executed by a practitioner | Hamstrings at slow velocity | MSV | HG |  | 0 | 0 | 0 | 0 | 1 | 1 |  |  |
| van der Krogt et al., 2016 |  | Passive stretching with controlled duration executed by a practitioner | Hamstrings at fast velocity | MSV | SG |  | 0 | 0 | 0 | 0 | 1 | 1 |  |  |
| van der Krogt et al., 2016 |  | Passive stretching with controlled duration executed by a practitioner | Hamstrings at fast velocity | MSV | HG |  | 0 | 0 | 0 | 0 | 1 | 1 |  |  |
| Falisse et al., 2018 | R² RMSE | Passive stretching with controlled duration executed by a practitioner | Hamstrings at fast velocity | MO1: MFL/MSV | SG | 2 | 0 | 0 | 0 | 0 | 0 | 0 | 0 | **0** |
| Falisse et al., 2018 |  | Passive stretching with controlled duration executed by a practitioner | Gastrocnemii at fast velocity | MO1: MFL/MSV | SG |  | 0 | 0 | 0 | 0 | 0 | 0 |  |  |
| Falisse et al., 2018 |  | Passive stretching with controlled duration executed by a practitioner | Hamstrings at fast velocity | MO3: MF/MFR | SG | 2 | 1 | 1 | 0 | 0 | 0 | 2 | 3 | **1.5** |
| Falisse et al., 2018 |  | Passive stretching with controlled duration executed by a practitioner | Gastrocnemii at fast velocity | MO3: MF/MFR | SG |  | 0 | 1 | 0 | 0 | 0 | 1 |  |  |
| Falisse et al., 2018 |  | Passive stretching with controlled duration executed by a practitioner | Hamstrings at fast velocity | MO2: MFLVA | SG | 2 | 0 | 0 | 0 | 0 | 0 | 0 | 0 | **0** |
| Falisse et al., 2018 |  | Passive stretching with controlled duration executed by a practitioner | Gastrocnemii at fast velocity | MO2: MFLVA | SG |  | 0 | 0 | 0 | 0 | 0 | 0 |  |  |
| Shin et al., 2020 | VAF R² RMSE | Passive stretching at controlled velocity using a device | Plantar flexors at slow velocity | MFL/MSV | SG | 3 | 1 | 0 | 0 | 1 | 1 | 3 | 9 | **3** |
| Shin et al., 2020 |  | Passive stretching at controlled velocity using a device | Plantar flexors at slow velocity | MFL/MSV | HG |  | 1 | 0 | 0 | 1 | 1 | 3 |  |  |
| Shin et al., 2020 |  | Passive stretching at controlled velocity using a device | Plantar flexors at fast velocity | MFL/MSV | SG |  | 1 | 0 | 0 | 1 | 1 | 3 |  |  |
| De Vlugt et al., 2011 | VAF | Passive stretching at controlled duration with an external device | Plantar flexors rotation duration of 2 seconds | Not specified | SG | 8 | 1 | 0 | 0 | 0 | 1 | 2 | 16 | **2** |
| De Vlugt et al., 2011 |  | Passive stretching at controlled duration with an external device | Plantar flexors rotation duration of 2 seconds | Not specified | HG |  | 1 | 0 | 0 | 0 | 1 | 2 |  |  |
| De Vlugt et al., 2011 |  | Passive stretching at controlled duration with an external device | Plantar flexors rotation duration of 1 second | Not specified | SG |  | 1 | 0 | 0 | 0 | 1 | 2 |  |  |
| De Vlugt et al., 2011 |  | Passive stretching at controlled duration with an external device | Plantar flexors rotation duration of 1 second | Not specified | HG |  | 1 | 0 | 0 | 0 | 1 | 2 |  |  |
| De Vlugt et al.,2011 |  | Passive stretching at controlled duration with an external device | Plantar flexors rotation duration of 0.5 second | Not specified | SG |  | 1 | 0 | 0 | 0 | 1 | 2 |  |  |
| De Vlugt et al., 2011 |  | Passive stretching at controlled duration with an external device | Plantar flexors rotation duration of 0.5 second | Not specified | HG |  | 1 | 0 | 0 | 0 | 1 | 2 |  |  |
| De Vlugt et al., 2011 |  | Passive stretching at controlled duration with an external device | Plantar flexors rotation duration of 0.25 second | Not specified | SG |  | 1 | 0 | 0 | 0 | 1 | 2 |  |  |
| De Vlugt et al., 2011 |  | Passive stretching at controlled duration with an external device | Plantar flexors rotation duration of 0.25 second | Not specified | HG |  | 1 | 0 | 0 | 0 | 1 | 2 |  |  |
| Koo; Mak, 2006 | Not reported | Passive stretching at controlled velocity using a device | Elbow extension | MFL/MSV | SG | 1 | 0 | 0 | 0 | 1 | 0 | 1 | 1 | **1** |
| Wang et al.,  2017 | VAF R² | Passive stretching at controlled velocity using a device | Wrist flexors at slow velocity | JAP/JAV | SG | 3 | 1 | 1 | 0 | 1 | 1 | 4 | 12 | **4** |
| Wang et al.,  2017 |  | Passive stretching at controlled velocity using a device | Wrist flexors at slow velocity | JAP/JAV | HG |  | 1 | 1 | 0 | 1 | 1 | 4 |  |  |
| Wang et al.,  2017 |  | Passive stretching at controlled velocity using a device | Wrist flexors at fast velocity | JAP/JAV | SG |  | 1 | 1 | 0 | 1 | 1 | 4 |  |  |
| Wang; Gäverth; Herman, 2018 | VAF | Passive stretching at controlled velocity using a device | Wrist flexors at slow velocity - Pre BoNT-A | JAP/JAV | SG | 6 | 1 | 0 | 0 | 1 | 1 | 3 | 18 | **3** |
| Wang; Gäverth; Herman, 2018 |  | Passive stretching at controlled velocity using a device | Wrist flexors at slow velocity - 4 weeks post BoNT-A | JAP/JAV | SG |  | 1 | 0 | 0 | 1 | 1 | 3 |  |  |
| Wang; Gäverth; Herman, 2018 |  | Passive stretching at controlled velocity using a device | Wrist flexors at slow velocity - 12 weeks post BoNT-A | JAP/JAV | SG |  | 1 | 0 | 0 | 1 | 1 | 3 |  |  |
| Wang; Gäverth; Herman, 2018 |  | Passive stretching at controlled velocity using a device | Wrist flexors at fast velocity - Pre BoNT-A | JAP/JAV | SG |  | 1 | 0 | 0 | 1 | 1 | 3 |  |  |
| Wang; Gäverth; Herman, 2018 |  | Passive stretching at controlled velocity using device | Wrist flexors at fast velocity - 4 weeks post BoNT-A | JAP/JAV | SG |  | 1 | 0 | 0 | 1 | 1 | 3 |  |  |
| Wang; Gäverth; Herman, 2018 |  | Passive stretching at controlled velocity using a device | Wrist flexors at fast velocity - 12 weeks post BoNT-A | JAP/JAV | SG |  | 1 | 0 | 0 | 1 | 1 | 3 |  |  |
| He; Norling; Wang, 1997 | Not reported | Pendulum test | Knee Joint | MFL/MSV | SG | 1 | 0 | 0 | 0 | 0 | 0 | 0 | 0 | **0** |
| He, 1998 | Not reported | Pendulum test | Knee Joint | MFL/MSV | SG | 1 | 0 | 0 | 0 | 0 | 0 | 0 | 0 | **0** |
| Fee; Foulds,  2004 | Not reported | Pendulum test/ Active model | Knee Joint | JAV | SG | 4 | 0 | 0 | 0 | 0 | 1 | 1 | 4 | **1** |
| Fee; Foulds,  2004 |  | Pendulum test/ Active model | Knee Joint | JAV | HG |  | 0 | 0 | 0 | 0 | 1 | 1 |  |  |
| Fee; Foulds,  2004 | Not reported | Pendulum test / Feedback from muscle stretching velocity model | Knee Joint | JAV | SG |  | 0 | 0 | 0 | 0 | 1 | 1 |  |  |
| Fee; Foulds,  2004 | Not reported | Pendulum test / Feedback from muscle stretching velocity model | Knee Joint | JAV | HG |  | 0 | 0 | 0 | 0 | 1 | 1 |  |  |
| Kim; Eom; Hase, 2011 | RMSE | Pendulum test | Knee Joint | JAV | SG | 1 | 0 | 1 | 0 | 0 | 1 | 2 | 2 | **2** |
| Le Cavorzin et al., 2001 | Not reported | Pendulum test | Knee Joint | JAP/JAV | SG | 2 | 0 | 0 | 0 | 0 | 1 | 1 | 2 | **1** |
| Le Cavorzin et al., 2001 |  | Pendulum test | Knee Joint | JAP/JAV | HG |  | 0 | 0 | 0 | 0 | 1 | 1 |  |  |
| De Groote et al.,  2018 | Not reported | Pendulum test | Knee Joint | JAP/JAV | SIM | 1 | 0 | 0 | 0 | 1 | 1 | 2 | 2 | **2** |
| De Groote et al.,  2018 |  | Pendulum test | Knee Joint | JT/TR | SIM | 1 | 0 | 0 | 0 | 1 | 1 | 2 | 2 | **2** |
| Feng; Mak,  1988 | Not reported | Pendulum test | Elbow joint | MFL/MSV | SG | 2 | 0 | 0 | 0 | 0 | 0 | 0 | 0 | **0** |
| Feng; Mak,  1988 |  | Pendulum test | Elbow joint | MFL/MSV | HG |  | 0 | 0 | 0 | 0 | 0 | 0 |  |  |
| Falisse et al.,  2018 | CCC | Gait (Hamstrings) | Knee and ankle - Hamstrings | MO1: MFL/MSV | SG | 2 | 0 | 0 | 0 | 0 | 0 | 0 | 2 | **1** |
| Falisse et al.,  2018 |  | Gait (Gastrocnemii) | Knee and ankle - Gastrocnemii | MO1: MFL/MSV | SG |  | 1 | 1 | 0 | 0 | 0 | 2 |  |  |
| Falisse et al.,  2018 |  | Gait (Hamstrings) | Knee and ankle - Hamstrings | MO3: MF/MFR | SG | 2 | 1 | 1 | 0 | 0 | 0 | 2 | 4 | **2** |
| Falisse et al.,  2018 |  | Gait (Gastrocnemii) | Knee and ankle - Gastrocnemii | MO3: MF/MFR | SG |  | 1 | 1 | 0 | 0 | 0 | 2 |  |  |
| Falisse et al.,  2018 |  | Gait (Hamstrings) | Knee and ankle - Hamstrings | MO2: MFLVA | SG | 2 | 0 | 0 | 0 | 0 | 0 | 0 | 2 | **0** |
| Falisse et al.,  2018 |  | Gait (Gastrocnemii) | Knee and ankle - Gastrocnemii | MO2: MFLVA | SG |  | 0 | 0 | 0 | 0 | 0 | 0 |  |  |
| Jansen et al.,  2014 | Not reported | Gait | Hip, Knee and Ankle | MFL/MSV | SIM | 1 | 0 | 0 | 0 | 0 | 0 | 0 | 0 | **0** |
| Falisse et al.,  2020 | R² CCC | Gait (Personalized Parameters) | Hip, Knee and Ankle | MF/MFR | SG | 1 | 1 | 1 | 1 | 0 | 0 | 3 | 3 | **3** |
| Falisse et al.,  2020 |  | Gait | Hip, Knee and Ankle | MF/MFR | SG | 1 | 1 | 1 | 0 | 0 | 0 | 2 | 2 | **2** |
| Bruel et al.,  2022 | Not reported | Gait | Hip, Knee and Ankle | MFL/MSV | SIM | 1 | 0 | 0 | 0 | 1 | 1 | 2 | 2 | **2** |
| Veerkamp et al., 2023 | R²  RMSE | Gait – Cluster 1 | Hip, Knee and Ankle | MO1:  MSV | SG | 3 | 0 | 0 | 0 | 0 | 0 | 0 | 0 | **0** |
| Veerkamp et al., 2023 |  | Gait – Cluster 2 | Hip, Knee and Ankle | MO1:  MSV | SG |  | 0 | 0 | 0 | 0 | 0 | 0 | 0 |  |
| Veerkamp et al., 2023 |  | Gait – Cluster 3 | Hip, Knee and Ankle | MO1:  MSV | SG |  | 0 | 0 | 0 | 0 | 0 | 0 | 0 |  |
| Veerkamp et al., 2023 |  | Gait – Cluster 1 | Hip, Knee and Ankle | MO2: MF | SG | 3 | 0 | 0 | 0 | 0 | 0 | 0 | 0 | **0** |
| Veerkamp et al., 2023 |  | Gait – Cluster 2 | Hip, Knee and Ankle | MO2: MF | SG |  | 0 | 0 | 0 | 0 | 0 | 0 | 0 |  |
| Veerkamp et al., 2023 |  | Gait – Cluster 3 | Hip, Knee and Ankle | MO2: MF | SG |  | 0 | 0 | 0 | 0 | 0 | 0 | 0 |  |

*Note.* The table presents the physiological plausibility score of the developed models according to the investigated tasks, target muscles or joints, and evaluated groups. **Types of feedback model**: **MSV:** Muscle stretching velocity; **MFL/MSV:** Muscle fiber length and muscle stretching velocity; **MF/MFR:** Muscle force and muscle force rate; **MFLVA:** Muscle fiber length, muscle stretching velocity, and muscle stretching acceleration**; JAP/JAV:** Joint angular position and joint angular velocity; **JAV:** Joint angular velocity; **JT/TR:** Joint torque and joint torque rate; **MF/MFR:** Muscle force and muscle force rate; **MF:** Muscle force. **NF:** Normalization factor. **Evaluated Groups:** SG: Spasticity group; HG: Healthy group; SIM: Computer simulation. **MO** (1, 2, and 3) refer to the models used in the studies.
