# Supplementary material for: Neuromusculoskeletal modeling of spasticity: A scoping review
Source: PLoS One. 2025 May 14;20(5):e0320153. doi: 10.1371/journal.pone.0320153 (PMC12077711; doi:10.1371/journal.pone.0320153)
Supplement: S4 Table — (DOCX) [file pone.0320153.s004.docx]

**S4 Table. Characteristics of the included studies concerning funding and competing interests.**

| **Studies** | **Report on the sources of funding** | **Conflict of Interest** |
| --- | --- | --- |
| He, Norling and Wang (1997) | Not reported | Not reported |
| He (1998) | Not reported | Not reported |
| Feng and Mak (1998) | Supported by the Research Committee of The Hong Kong Polytechnic University. | Not reported |
| Le Cavorzin et al. (2001) | Not reported | Not reported |
| Fee and Foulds (2004) | Not reported | Not reported |
| Koo and Mak (2006) | Supported by the Research Committee of the Hong Kong Polytechnic University. | Not reported |
| De Vlugt et al. (2010) | This study was performed as part of the Dutch TREND project (Trauma RElated Neuronal Dysfunction), supported by the Dutch Government (grant nr. BSIK03016). | The authors declared that they had no competing interests. |
| Kim, Eom and Hase (2011) | This work was supported by a grant of the Korea Healthcare technology R&D Project, Ministry for Health & Welfare, Republic of Korea (A084177). | Not reported |
| Jansen et al. (2014) | Funded by a grant of the KU Leuven Research council (IDO/07/012), the Research Foundation, Flanders (G.0395.09), the Flemish research council (KN 1.5.017.08) and by the Hercules Foundation (HER/09/030). | The authors declared that they had no competing interests. |
| van der Krogt et al. (2016) | Funded by NCSRR Visiting Scholar and Pilot Project Program grants to MK, supported by NIH research infrastructure grant R24 HD065690. LB was supported by PhD grant DBOF/12/058 and FWO post-doc grant 12R4215N. None of these funding sources had any role in the design of the study, the collection, analysis, and interpretation of data, nor in writing the manuscript. | The authors declared that they had no competing interests. |
| Wang et al. (2017) | Supported by the Stockholm Brain Institute and Promobilia Foundation. | We declare a potential conflict of interest as follow: the NFmethod described in this paper has been patented by A. Fagergren (WO/2008/121,067). Author J. Gäverth owns part of the commercial rights of the measurement instrument described in this study as shareholders of the manufacturing company Aggero MedTech AB. |
| Wang, Gäverth and Herman (2018) | Supported by Promobilia Foundation, Norrbacka-Eugeniastiftelsen, and Carl Tryggers Stiftelsen | Author JG owns part of the commercial rights of the measurement instrument described in this study as shareholders of the manufacturing company Aggero MedTech AB. The remaining authors declare that the research was conducted in the absence of any commercial or financial relationships that could be construed as a potential conflict of interest. |
| Falisse et al. (2018) | All authors were supported by the IWTTBM grant Sim-CP (140184) (www.vlaio.be). AF also received a Ph.D. grant (1S35416N) of the Research Foundation Flanders (FWO, www.fwo. be). LB also received a postdoctoral grant (12R4215N) of the Research Foundation Flanders (FWO, www.fwo.be) and a grant (016.186.144) | The authors declared that they had no competing interests. |
| De Groote et al. (2018) | This work was supported by the National Institute for Child Health and Human Development (https://www.nichd.nih.gov/Pages/index.aspx) grants R01 HD46922 and R01 HD090642 to LHT, the National Institute of Neurological Disorders and Stroke (https://www.ninds.nih.gov) F31NS093855 fellowship to KPB, Banting Postdoctoral Fellowship (http://bantingresearchfoundation.ca/) to BCH, and Research Foundation – Flanders (http://www.fwo.be/) travel grant V436017N and KU Leuven (https://www.kuleuven.be/onderzoek/ondersteuning/if) start-up grant STG/16/032 to FDG. The funders had no role in study design, data collection and analysis, decision to publish, or preparation of the manuscript. | The authors declared that they had no competing interests. |
| Shin et al. (2020) | Not reported | Not reported |
| Falisse et al. (2020) | This work was supported by the IWT-TBM grant SimCP (140184). AF also received a Ph.D. grant (1S35416N) from the Research Foundation Flanders (FWO). HK received a H2020-MSCA individual fellowship (796120). LB-O received a postdoctoral grant (12R4215N) from the Research Foundation Flanders (FWO) and a grant (016.186.144) from the Netherlands Organization for Scientific Research (NWO). | The authors declared that they had no competing interests. |
| Bruel et al. (2022) | Open access funding provided by Ecole Polytechnique Federale de Lausanne. This work was supported by the SimGait Sinergia project funded by the Swiss National Science Foundation, grant agreement No. 177179, https://p3.snf.ch/project-177179. A.I., Pr S.A. and A.D.R. received this grant. | The authors declared that they had no competing interests. |
| Veerkamp et al. (2023) | Not reported | Not reported |
